# Immunomodulatory Effects of SGLT2 Inhibitors and Metformin in Managing Rheumatic Diseases: A Narrative Review

**DOI:** 10.31138/mjr.010324.ies

**Published:** 2024-09-30

**Authors:** Naveenkumar Nallathambi, Rahul Bisaralli, Mahabaleshwar Mamadapur3

**Affiliations:** 1 Institute of Internal Medicine, Madras Medical College, Chennai, Tamilnadu, India; 2 Department of Clinical Immunology and Rheumatology, SDM College of Medical Sciences and Hospital, Dharwad, Karnataka, India

**Keywords:** rheumatic disease, immunomodulatory effect, Type 2 diabetes mellitus, metformin, sodium-glucose cotransporter-2 (SGLT2) inhibitors

## Abstract

Limited studies summarise the immunomodulatory effects of SGLT2 inhibitors and Metformin in managing rheumatic diseases. The present narrative review aims to fill this knowledge gap by gathering information based on existing clinical evidence. A narrative review was conducted in November 2023 to identify studies investigating the impact of SGLT2 inhibitors and Metformin on rheumatic diseases. A literature search was performed across primary databases, including Medline/PubMed, Scopus, and Web of Science. Supplementary sources like Google Scholar, PubMed Central, Cochrane Library, and ScienceDirect were also consulted. Studies were screened for relevance, and those lacking pertinent outcome data were excluded. The review corroborates the multifaceted potential of Metformin as an adjunctive therapy in autoimmune rheumatologic conditions, offering avenues for further exploration and clinical application to enhance patient outcomes. However, limited literature exists on the clinical effects of SGLT2 inhibitors in rheumatic diseases.

## INTRODUCTION

Rheumatic diseases are a growing public health concern due to their rising prevalence and economic burden on individuals and healthcare systems. The long-term morbidity, disability, and loss of productivity associated with these conditions also impair quality of life. The aging global population further exacerbates this disease burden.^1^ The prevalence of locomotor disability increases with age, from 3.1% in those under 60 to around 50% in those over 75, with one-third of cases having rheumatologic problems.^2^ The interconnection between diabetes mellitus (DM) and rheumatic diseases extends to their shared pathobiology and treatment modalities, influencing the risk of developing DM and glycemic management.^3^ This bidirectional relationship between DM and rheumatic diseases is further compounded by poorer treatment outcomes and an increased likelihood of complications, notably infections, attributable to the combined impact of DM and immunosuppressant therapy.^3^ In a comprehensive systematic review and meta-analysis involving 1,629,854 patients diagnosed with rheumatoid arthritis (RA), findings revealed an increased incidence of DM among individuals with RA.^4^ Rheumatic diseases often involve renal complications, encompassing vascular, glomerular, and tubulointerstitial alterations.^5^ According to Hill et al., the prevalence of stage 3 chronic kidney disease (CKD) or lower (characterised by a glomerular filtration rate [GFR] of 60 mL/min or less) among patients in rheumatology clinics is approximately 18%. This figure starkly contrasts with the mere 5% prevalence observed within the general population.^6^ Patients with autoimmune rheumatic diseases such as RA and systemic lupus erythematosus (SLE) face an increased risk of cardiovascular disease, including premature atherosclerosis.^7,8^ Heart failure stands as a principal contributor to cardiovascular mortality in individuals diagnosed with RA. Research indicates a notable elevation in the occurrence of heart failure with preserved ejection fraction (HFpEF) among RA patients compared to their non-RA counterparts.^9,10^

Rheumatic diseases cause long-term disability and account for 15–45% of primary care consultations due to musculoskeletal diseases. A study based in Italy found that inflammatory rheumatic diseases had a negative impact on quality of life. RA had the most considerable effect, followed by fibromyalgia, osteoporosis-induced vertebral fractures, hip osteoarthritis (OA), and systemic sclerosis.^11^ Considering the current treatment objectives, namely treat-to-target approach and remission, more personalised and effective treatments are warranted for effective management.^12^ Some RA patients do not respond well to newer treatments, especially in low-income countries with limited access to biological DMARDs. Repurposing drugs like Metformin, sodium-glucose cotransporter 2 (SGLT2) inhibitors, dipeptidyl peptidase 4 (DPP-4) inhibitors, glucagon-like peptide 1 (GLP-1) receptor agonists, insulin and its analogs, thiazolidinediones, and sulfonylureas has been suggested as an effective strategy to meet the unmet therapeutic needs in rheumatology.^13^

SGLT2 inhibitors are oral hypoglycemic medications primarily indicated for the management of type 2 diabetes mellitus (T2DM). Dapagliflozin, canagliflozin, ertugliflozin, and empagliflozin are the four SGLT2 inhibitors approved by the US Food Drug Administration.^14^ SGLT2 inhibitors have potential benefits such as reduced cardiovascular risk and prevention of end-stage renal disease. They hold promise for managing autoimmune rheumatic diseases.^15^

Metformin, a first-line treatment for T2DM, has immunomodulatory and anti-inflammatory effects. Studies have shown that it can be beneficial in managing synovitis and joint destruction and mitigating cartilage degradation in patients with RA and OA. It can also enhance immunomodulatory, anti-inflammatory, and chondroprotective effects when used as an adjunctive therapy to traditional treatments.^16^

Since limited studies summarise the immunomodulatory effects of SGLT2 inhibitors and Metformin in managing rheumatic diseases, the present narrative review has attempted to fill the knowledge gap based on existing clinical evidence.

## METHODOLOGY

### Search strategy

A narrative review was conducted in November 2023 to identify pertinent studies investigating the impact of SGLT2 inhibitors and Metformin on rheumatic diseases. A literature search was performed using search terms such as “SGLT2 AND Immunomodulatory effect”, “Metformin AND Immunomodulatory effect”, “SGLT2 inhibitors AND Rheumatic diseases”, “Metformin AND Rheumatic diseases”, and relevant variations, sςuch as outlined in **Supplementary Table 1**. Initial searches were conducted across primary databases, including Medline/PubMed, Scopus, and Web of Science, to ensure a comprehensive exploration of the topic. Additionally, supplementary sources such as Google Scholar, PubMed Central, Cochrane Library, and ScienceDirect were consulted to validate findings and identify any overlooked relevant studies. Furthermore, select review articles were cross-referenced to procure additional references, enriching the breadth of the literature examined.

### Screening of the studies

The search results from Medline/PubMed, Scopus, and Web of Science were imported into the reference management tool EndNote. Additionally, studies retrieved from supplementary sources like Google Scholar, PubMed Central, and Science-Direct underwent title screening before being incorporated into the reference management tool. Subsequent screening involved evaluating abstracts and full texts to exclude reviews, editorials, duplicates, non-English studies, and those lacking pertinent outcome data from further consideration.

## IMMUNOMODULATORY AND CLINICAL EFFECTS OF METFORMIN IN AUTOIMMUNE RHEUMATOLOGIC CONDITIONS

### Rheumatoid arthritis

#### Preclinical studies

Kang et al. noted that Metformin significantly reduced arthritis severity in collagen-induced arthritis (CIA) murine model by reducing Th17 cell differentiation through AMP-activated protein kinase (AMPK) activation, leading to downregulation of mammalian target of rapamycin complex 1 (mTORC1) and signal transducer and activator of transcription-3 (STAT3).^17^ A CIA murine model study by Son et al. reported that Metformin was shown to decrease the severity of arthritis, improve Treg cell development, limit osteoclast generation, and suppress differentiation of Th17 cells.^18^ Yan et al. found that Metformin reduced the arthritis severity and the inflammatory cytokine generation by increasing autophagic flux and inhibiting the nuclear factor kappa-light-chain-enhancer of activated B cell (NF-κB) pathway.^19^ According to Kim et al., Metformin demonstrated therapeutic efficacy in obese CIA mice by enhancing the production of pAMPK and fibroblast growth factor 21. Furthermore, metformin-induced brown adipose tissue (BAT) differentiation, consequently enhancing a reciprocal balance between T helper (Th) 17 and regulatory T (Treg) cells both in vitro and in vivo **(Table 1)**.^20^

**Table 1.** Immunomodulatory effects of Metformin in RA.

**Author, year**	**Disease**	**Experimental setting**	**Study findings (effects of Metformin)**
Kang et al., 2013^9^	Autoimmune arthritis	CAIA murine model	Arthritis severity reduction and Th17 cell differentiation inhibition
Son et al., 2014^10^	Autoimmune arthritis	CIA murine model	Suppression of Th17 cell differentiation and development of Treg cell differentiation
Yan et al., 2015^11^	Arthritis	K/BxN arthritis model	Inflammatory cytokine production suppression, autophagic flux development, and NF-κB pathway suppression
Kim et al., 2018^12^	Autoimmune arthritis	CIA mice model	Brown adipose tissue differentiation and FGF21 expression production
Chen et al., 2019^13^	RA	RA synovial tissue	RA-FLS proliferation inhibition via IGF-IR/PI3K/AKT/m-TOR pathway
Chen et al., 2020^14^	RA	RA synovial tissue	Inhibition of RA FLS proliferation and migration
Fan et al., 2020^15^	Arthritis	CIA mice model	Attenuated bone and cartilage destruction, and synovial inflammation
Matsuoka et al., 2021^16^	RA	RA synovial fibroblast cells stimulated with TNF-α	Osteoclastogenesis, angiogenesis and inflammatory response inhibition
Park et al., 2021^17^	Arthritis	CIA mice model	Th17 differentiation and IL-6 signaling suppression
El-Sayyad et al., 2021^19^	Arthritis	Arthritis murine model	Anti-inflammatory effects
Gallagher et al., 2020^20^	RA	*in vitro* RA FLS cultures and *ex vivo* RA synovial explant cultures	Inflammatory cytokines production suppression
Kim et al., 2020^18^	RA	CIA mice model	Th17 and Treg cell balance regulation, and improvement in arthritis severity and metabolic profile
Makar et al., 2020^21^	RA	RA-induced rat model	Improvement in RA parameters
Yang et al., 2017^23^	Arthritis	CIA rat	Th17/Treg cells balance regulation, mTOR inhibition, and AMPK activation
Jhun et al., 2016^22^	Autoimmune arthritis	CIA mice model	Reduction of IL-17 expression, Th17 cells, and induction of Treg cell production

CAIA: Collagen antibody-induced arthritis; CIA: Collagen-induced arthritis; RA: Rheumatoid arthritis; RA-FLS: rheumatoid arthritis fibroblast-like synoviocytes; TNF: Tumor necrosis factor; FGF: fibroblast growth factor 21; NF-κB: Nuclear factor kappa B; AMPK: Adenosine monophosphate-activated protein kinase; IL: Interleukin; mTOR: mechanistic target of rapamycin; IGF-IR: Insulin-like growth factor-IR; PI3K: phosphoinositide 3-kinase; AKT: protein kinase B.

Chen et al. found that Metformin inhibits human rheumatoid arthritis synovial fibroblasts (RA-FLS) proliferation by inducing cell cycle arrest and regulating p70S6K and 4E-BP1 phosphorylation. Additionally, Metformin induces G2/M arrest in RA-FLS via inhibition of the IGF-IR/PI3K/AKT pathway.^21^ Chen et al. corroborated that Metformin, functioning as an activator of AMPK, effectively prevented the proliferation and migration of RA-FLSs.^22^ Fan et al. demonstrated that metformin treatment reduced synovial hyperplasia, inflammation, neovascularisation, and expression of matrix-degrading enzymes in the cartilage of CIA rats.^23^ Another study on tumour necrosis factor (TNF)-α stimulated RA-FLS MH7A cells demonstrated that Metformin inhibited osteoclastogenesis and decreased the expression of genes unique to osteoclasts. Metformin inhibited the expression of growth factor, protease, and inflammatory cytokine genes in MH7A cells stimulated by TNF-α (Matsuoka et al.).^24^ A combination of Metformin and LMT-28 was more effective in reducing arthritis severity in mice. The therapy suppressed the differentiation of Th17 cells while enhancing the differentiation of Treg cells and showed inhibitory effects on interleukin (IL)-6 signalling (Park et al.).^25^ Kim et al. found that combining rapamycin and Metformin reduced arthritis and inflammation without causing mitochondrial dysfunction. The combination improved the metabolic profile, reduced clinical arthritis score and histological inflammation, and enhanced the balance between T helper 17 and regulatory T cells in mice.^26^ El-Sayyad et al. found that combining Metformin and omega-3 can effectively modulate several signalling pathways, including miRNA-155, 146a, and 34. These pathways play crucial roles in the development of RA. This combination therapy offers a significantly improved additive anti-inflammatory response comparable to the effectiveness of methotrexate.^27^ Gallagher et al. revealed that Metformin promotes the resolution of synovial inflammation in RA, partly through the phosphorylation of AMPK. Metformin can modify cellular metabolic activity, leading to a notable decrease in the oxidative phosphorylation/glycolysis ratio, indicating a shift towards glycolytic metabolism **(Table 1)**.^28^ Makar et al. showed in a rat model of RA that Metformin, alone or with methotrexate, improved rheumatoid factor, C-reactive protein (CRP), reduced glutathione, (TNF)-α, arthritis score, and histological RA-related alterations.^29^ In a CIA murine model study, combining Metformin and CoQ10 led to more significant improvements in arthritis and mitochondrial function compared to individual treatments. This was linked to reduced IL-17 expression, decreased Th17 cells, and increased Treg cell induction, indicating an enhanced anti-inflammatory response (Jhun et al.).^30^ In a CIA rat model study, Metformin reduced hind paw swelling, inhibited excessive synovial tissue growth, and lowered proinflammatory cytokine levels in the bloodstream. Metformin influenced synovial tissue by upregulating Bax and cleaved-Caspase-3 expression, downregulating Bcl2, and activating AMPK while inhibiting mTOR in both the spleen and synovium (Yang et al.) **(Table 1)**.^31^ A two-sample Mendelian randomisation study by Liang et al. reported a negative causal association between RA and metformin treatment, suggesting that metformin therapy may inhibit RA pathogenesis.^32^

#### Clinical studies

*Randomised controlled trials (RCTs):* A randomised trial by Abdallah et al. with 120 active RA patients found that those receiving Metformin and methotrexate achieved higher rates of American College of Rheumatology (ACR)20 response and Disease Activity Score (DAS) remission after 12 weeks. Significant improvements were also observed in DAS28-3 (CRP), ACR50, ACR70, and Health Assessment Questionnaire–Disability Index (HAQ-DI).^33^ In a study involving 66 RA patients on csDMARDs, Gharib et al. observed that adding Metformin significantly lowered serum CRP and adiponectin levels, reduced disease activity, and improved patients’ quality of life **(Table 3)**.^34^

**Table 3.** Clinical studies on effects of Metformin in autoimmune rheumatologic conditions.

**Author, year**	**Disease**	**Study setting**	***Study findings (effects of Metformin)***
Hao et al., 2018^54^	SLE	Patients with SLE	Reduction in glucocorticoid dosage, restoration of Th17/Treg cells balance
Sun et al., 2020^55^	SLE	Patients with SLE	Reduction in disease flares
Wang et al., 2015^56^	SLE	Patients with SLE	Decreased prednisone exposure, clinical flares
Lu et al., 2019^24^	RA	Patients with RA and T2DM	Decreases admission rates of patients with RA and DM
Abdallah et al., 2021^25^	RA	Patients with RA	Elevated ACR20 response achieving rate and DAS remission achieving rate
Naffaa et al., 2020^26^	RA	Patients who initiated therapy with Metformin	Decreased risk of incident RA
Gharib et al., 2021^27^	RA	Patients with RA	Reduced disease activity, serum C reactive protein, and adiponectin levels
Zhang et al., 2023^29^	RA	Patients with RA	Decreased VAS scores, CD4+/CD8+ ratio, and CD4+ expression
Wang et al., 2021^63^	Sjögren's syndrome	Patients with T2DM	Decreased risk for SS
Veenstra et al.; 2022	Gout	Patients with Gout and T2DM	At six months, 62.8% of patients attained the target sUA level

SLE: Systemic lupus erythematosus, SS: Sjogren›s syndrome, T2DM: Type 2 diabetes mellitus; ACR: American College of Rheumatology; CD: Cluster of differentiation; VAS: visual analog scale; sUA: Serum uric acid.

*Observational studies:* In a study involving 1268 RA and T2DM patients, Lu et al. observed that administering Metformin with cyclooxygenase-2 (COX-2) inhibitors lowered admission rates compared to COX-2 inhibitors alone.^35^ In an 18-year cohort study involving 113,749 adult metformin users, Naffaa et al. found that treatment adherence was associated with decreased RA risk. Patients with 40–59% follow-up days coverage had the lowest risk compared to non-adherent individuals **(Table 3)**.^36^ In a study comprising 124 RA patients, those in the metformin group (Metformin + conventional therapy) showed significantly lower VAS scores, CD4+/CD8+ ratio, and CD4+ expression compared to those in the conventional therapy group after 90 days of treatment (Zhang et al.) **(Table 3)**.^37^

### Systemic lupus erythematosus

#### Preclinical studies

Yin et al. found increased glycolysis and mitochondrial oxidative metabolism in CD4+ T cells from lupus-prone mice and systematic lupus erythematosus (SLE) patients, both ex vivo and after in vitro activation, compared to nonautoimmune controls. Treating mice with Metformin and 2DG normalised T-cell metabolism, restored CD4+ T function, and reversed disease phenotypes, including autoantibody production and renal disease.^38^ In a study conducted on peripheral blood mononuclear cells (PBMCs) of lupus patients, Metformin significantly reduced P-gp expression and proinflammatory cytokines. It elevated the anti-inflammatory cytokine IL-10 production (Kansurkar et al).^39^ In a murine lupus model induced by pristane, both oral and intraperitoneal metformin groups exhibited decreased levels of interferon (IFN)-γ compared to saline controls (Sumantri et al.) **(Table 2)**.^40^

**Table 2.** Immunomodulatory effects of Metformin in other rheumatic diseases.

**Author, year**	**Disease**	**Experimental setting**	***Study findings (effects of Metformin)***
Yin et al., 2015^49^	SLE	Lupus-prone mice model and CD4+ T cells from SLE patients	CD4+ T function restoration and lupus phenotype reversion
Kansurkar et al., 2018^50^	Lupus	PBMCs of lupus patients	Reduction in IL-1, IL-6, IFNγ and elevation in IL10 production
Sumantri et al., 2021^51^	SLE	SLE mice model	Reduction in IFNγ level
Jang et al., 2020^52^	SLE	Mice lupus model	Promoting the immunoregulatory effect of Ad-MSCs via STAT1 expression and improvement in disease activity
Chen et al., 2022^53^	Lupus nephritis	MRL/MpJ-Faslpr/J (MRL/lpr) mouse model	Suppression of inflammation and renal necroptosis by AMPK/STAT3 pathway
Qin et al., 2018^57^	Ankylosing spondylitis	Fibroblast cells from ankylosing spondylitis patients	Robust anti-osteogenic action on fibroblasts, Pi3k/Akt and AMPK pathway activation
Nascimento et al., 2023^61^	SS	SS-prone mouse models (IL14α Tg-mice)	Saliva secretion restoration and saliva secretion via Ca2+ signaling
Tsuji et al., 2020^64^	Psoriasis	Mouse psoriasis model, TNF-α and IL-17A stimulated normal human epidermal keratinocytes	preventing the activation of the NLRP3 inflammasome, reduction in epidermal hyperplasia, and inflammatory cell infiltration and ear thickness
Moon et al., 2021^65^	Scleroderma	Scleroderma mice model	Proinflammatory cytokines inhibition and mTOR-STAT3 signaling suppression
Kim et al., 2019^62^	SS	SS mice model	Reduction in hypofunction and inflammation of the salivary glands, effector T cells inhibition, regulatory T cells activation, B cell differentiation regulation
Wang et al., 2019^66^	Scleroderma	Scleroderma mice model	Regulation of Treg/Teff cells balance and spleen germinal center formation suppression.
Vazirpanah et al., 2019^67^	Gout	PBMCs from patients with gout	Suppression of mTOR signaling
Liang et al., 2023 ^28^	RA	Genome-wide association study data involving Patients with RA	Negative causality between RA and Metformin treatment

SLE: Systemic lupus erythematosus, SS: Sjogren›s syndrome, AMPK: Adenosine monophosphate-activated protein kinase, NHEKs: normal human epidermal keratinocytes, PBMCs: Peripheral blood mononuclear cells; CD: Cluster of differentiation; IL: Interleukin; IFNγ: Interferon-gamma; Ad-MSCs: Adipose-derived mesenchymal stem cells; STAT: Signal transducer and activator of transcription; NLRP3: NOD-like receptor protein 3.

Jang et al. demonstrated that Metformin enhanced the immunomodulatory properties of Ad-MSCs through STAT1 activation. Additionally, it was shown that the administration of metformin-treated Ad-MSCs significantly ameliorated the clinical features of lupus, renal inflammation, and immune cell dysfunction in MRL/lpr mice by regulating DNT, Th17, and Treg cells.^41^ In an experimental lupus nephritis (LN) mice model study by Chen et al., metformin treatment reduced renal damage, improved renal function, and increased the mice survival rate. The mechanism of action primarily involved AMPK-mediated STAT3 inhibition and necroptosis, suppressing both systemic and intrarenal inflammatory responses in the kidneys **(Table 2)**.^42^

#### Clinical studies

*RCTs:* In a study by Sun et al. involving 201 SLE patients, a significant reduction in flares was observed with Metformin compared to placebo at the 12th-month follow-up (21.2% vs 35.3%), particularly for serologically quiescent patients.^43^ A study by Wang et al. noted that metformin add-on therapy resulted in a reduction in prednisone exposure and clinical flares compared to the conventional treatment group **(Table 3)**.^44^

Observational studies: Hao et al. noted that administering a combination of Metformin and conventional therapy to 23 SLE patients reduced prednisone dosage and symptom alleviation. The combination effectively upregulated Treg cells and increased Th17, restoring the balance of Th17/Treg cells in SLE. Prednisone dosage decreased significantly from 23.70±19.41 mg/d at week 0 to 20.44±17.03 mg/d at week 6 **(Table 3)**.^45^

### Ankylosing spondylitis

#### Preclinical studies

An *in vitro* study by Qin et al. on fibroblasts from femoral neck fracture and ankylosing spondylitis (AS) patients showed that metformin administration effectively suppressed ossification and downregulated osteogenic-specific markers. Additionally, it reduced inflammatory marker levels **(Table 2)**.^46^

### Sjögren’s syndrome

#### Preclinical studies

Metformin treatment restored Ca2+ signalling in IL14α-transgenic mice with Sjögren’s syndrome (SS), preventing immune cell infiltration and restoring saliva secretion. Restoring Ca2+ signalling inhibited alarmin release and prevented endoplasmic reticulum stress activation (Nascimento et al.).^47^ Metformin effectively reduced salivary gland inflammation, restored salivary flow rate, and regulated Tfh and follicular regulatory T cell balance in an SS mice model. It also decreased the level of IL-6, TNF-α, and IL-17 mRNA and protein in the glands, reduced the numbers of Th17 and Th1 cells while increasing regulatory T cells in peripheral blood and spleen, and maintained the balance between IL-10- and IL-17-producing B cells by reducing B cell differentiation into germinal centre B cells (Kim et al.) **(Table 2)**.^48^

#### Clinical studies

Observational studies: A 13-year retrospective population-based study found that individuals with T2DM using Metformin had a significantly lower incidence of acquiring SS compared to non-metformin controls (16.82 vs 40.83 per 100,000 person-years) (Wang et al.) **(Table 3)**.^49^

### Psoriasis

#### Preclinical studies

Tsuji et al. demonstrated that metformin treatment suppressed inflammatory responses in psoriasis by blocking NLRP3 inflammasome activation. Oral metformin treatment also reduced inflammation in vivo. In a mouse model, metformin treatment decreased ear thickness, epidermal hyperplasia, and inflammatory cell infiltration while downregulating various cytokines **(Table 2)**.^50^

### Scleroderma

#### Preclinical studies

In a study by Moon et al., Metformin reduced the expression of proinflammatory factors and fibrosis-inducing molecules in a scleroderma mice model both in vivo and in vitro.65 Metformin was found to improve the effects of bleomycin in a mice model study by Wang et al. It reduced skin inflammation and fibrosis, restored balance between Treg and Teff cells, and minimised spleen germinal centre formation **(Table 2)**.^51^

### Gout

#### Preclinical studies

Vazirpanah et al. revealed dysregulated mTOR signalling in PBMCs of gout patients compared to healthy controls. Inhibiting mTOR with Metformin or rapamycin reduced cell death and inflammatory mediator release **(Table 2)**.^52^

#### Clinical studies

*Observational studies:* In a retrospective cohort study conducted by Veenstra et al. involving 160 patients with both gout and DM who were treated with Metformin, results demonstrated that after six months, 62.8% and 54.9% of participants in the metformin and control groups, respectively, achieved the target serum uric acid (sUA) levels. However, the authors were unable to ascertain a clinically significant anti-inflammatory or urate-lowering effect of Metformin among patients initiating urate-lowering therapy (ULT) while receiving standard care for flare prophylaxis.^53^

## IMMUNOMODULATORY AND CLINICAL EFFECT OF SGLT2 INHIBITORS IN RHEUMATIC DISEASES

### Systemic lupus erythematosus

#### Preclinical studies

Zhao et al. revealed that SGLT2 inhibitors have a renoprotective effect on lupus mice. According to the study, the SGLT2 inhibitor empagliflozin can alleviate podocyte injury by reducing mTORC1 activity, enhancing autophagy, and attenuating inflammation in lupus-prone MRL/lpr mice **(Table 4)**.^54^

**Table 4.** Effects of SGLT2 inhibitors in rheumatic diseases.

**Author, year**	**Disease**	**Study setting**	**Study findings (effects of SGLT2)**
Zhao et al., 2023^68^	Lupus nephritis	Lupus mice model, SLE patients	Inflammation reduction, Improved autophagy via mTORC1 activity suppression, proteinuria reduction, renal benefit
Jorge et al., 2023^69^	SLE	Patients with SLE	Reduced risk of renal progression and MACE
Lo et al., 2023^71^	SLE	Patients with systemic lupus erythematosus and comorbid T2DM	Reduced risk of hospitalisation, and renal and cardiovascular conditions
Petri et al., 2023^72^	SLE	Patients with SLE	eGFR decline reduction and urine protein/creatinine ratio improvement
Wang et al., 2022^70^	SLE	Patients with SLE	Adequate safety profile
Wei et al., 2023^74^	Gout	Patients with gout and T2DM	Reduced risk of recurrent gout flares and mortality
Fralick et al., 2020^75^	Gout	Patients with T2DM	Lower incidence of gout
Lund et al., 2021^76^	Gout	SGLT2i users with propensity score matched GLP1-RA users	
Chung et al., 2021^77^	Gout	Patients with T2DM	
Zhou et al., 2023^78^	Gout	Patients with T2DM	

ER: Endoplasmic reticulum; SLE: Systemic lupus erythematosus; T2DM: Type 2 diabetes mellitus; MACE: Major adverse cardiac events; SGLT2i: systemic lupus erythematosus inhibitors; GLP-1 RA: Glucagon-like peptide-1 receptor agonists, eGFR: estimated glomerular filtration rate.

#### Clinical studies

*Clinical trials:* Jorge et al. demonstrated the cardiac and renal benefits of SGLT2 inhibitors in SLE/LN patients utilising electronic health records and target trial emulation to compare the outcomes of SGLT2 inhibitors with DPP-4 inhibitors in SLE patients. Propensity match scoring revealed that SGLT2 inhibitor use was associated with a reduced risk of major cardiovascular events (HR: 0.69) and renal progression (eGFR decline by 30% or progression to ESRD; HR: 0.71) compared to DPP-4 inhibitors. Similar benefits were noted in lupus nephritis patients.^55^ Wang et al. conducted a phase I/II trial with dapagliflozin involving 38 SLE patients. The study showed satisfactory safety outcomes, reduced BMI by 0.50 kg/m2 and systolic blood pressure by 3.94 mm Hg, increased hemoglobin levels by 8.26 g/L, and stable eGFR overall. LN patients with baseline eGFR <90 mL/min/1.73 m2 showed improved eGFR slope **(Table 4)**.^56^

*Observational studies:* Zhao et al. evaluated nine LN patients treated with SGLT2 inhibitors for over two months, and significant proteinuria reduction (29.6% to 96.3%) was observed. Moreover, the estimated glomerular filtration rate (eGFR) remained stable throughout the treatment.^54^ Lo et al. showed that using SGLT2 inhibitors instead of DPP-4 inhibitors led to significantly lower risks of developing acute kidney injury, chronic/end-stage renal disease, hospitalisation, and heart failure.^57^ A study by Petri et al. involving 45 SLE patients observed that receiving SGLT2 inhibitors contributed to improved urine protein/creatinine ratio and less deterioration in eGFR **(Table 4)**.^58^

### Gout

#### Clinical studies

*Observational studies:* Wei et al. showed that SGLT2 inhibitors can reduce the risk of recurrent gout flares and mortality in patients with gout and T2DM compared to other treatments. This suggests that SGLT2 inhibitors help reduce the burden of gout flares and narrow the mortality gap between gout patients and the general population.59 In a study of 295,907 adults with T2DM, those prescribed SGLT2 inhibitors had a lower incidence of gout compared to those prescribed GLP1 agonists (4.9 vs 7.8 events per 1000 person-years) with a hazard ratio of 0.64 (Fralick et al.).^60^ Similar results were noted in three other studies by Lund et al., Chung et al., and Zhou et al., where SGLT2 inhibitors use was associated with lower risks of gout rate in T2DM patients.^61–63^ Lund et al. suggested that using SGLT2 inhibitor is associated with a lower risk of gout compared to GLP1 receptor agonists and DPP-4 inhibitors **(Table 4)**.^61^

## CLINICAL RELEVANCE OF LITERATURE EVIDENCE

The narrative review examined six articles that assessed the impact of metformin RA in animal models. The aggregated findings revealed that Metformin demonstrated anti-inflammatory effects across diverse murine models by reducing arthritis severity and modulating immune cell differentiation. Metformin’s immunomodulatory effects, particularly in enhancing regulatory T-cell development and suppressing Th17 cell differentiation, suggest a multifaceted approach to controlling RA-associated inflammation. Additionally, the studies underscored its diverse effects, including the reduction of arthritis severity, modulation of immune cell differentiation (Th17 and Treg cells), inhibition of inflammatory cytokine production, and protective effects against synovitis and cartilage degradation. Furthermore, the studies explored the potential synergistic benefits of combining Metformin with other agents such as LMT-28, rapamycin, and omega-3. The observed alterations in cellular metabolism and glucose regulation further support metformin’s broader therapeutic effects beyond direct immunomodulation.

For other rheumatologic diseases, the review considered five preclinical studies for SLE, two for scleroderma, three for SS, and one for LN, AS, psoriasis, and gout. The comprehensive review of studies highlights the diverse and promising therapeutic effects of Metformin across various autoimmune and inflammatory conditions. Metformin demonstrated promising outcomes in SLE by restoring T-cell metabolism and function and reducing disease phenotypes, including autoantibody production and renal disease. Similarly, in LN models, Metformin exerted beneficial effects by suppressing systemic and intrarenal inflammatory responses through AMPK-mediated pathways. Studies in murine models of SS highlighted Metformin’s ability to restore salivary gland function, reduce immune cell infiltration, and regulate cytokine production, suggesting its potential as a therapeutic agent for this condition. In AS, the drug showed anti-inflammatory effects by effectively suppressing ossification, thus offering potential avenues for disease management. Metformin exhibited anti-inflammatory effects in psoriasis by suppressing the inflammatory responses of keratinocytes and reducing the severity of skin inflammation. Furthermore, Metformin attenuated skin inflammation, fibrosis, and immune dysregulation in scleroderma models, suggesting its potential as a therapeutic agent for this condition. In gout, the drug demonstrated promising results by inhibiting mTOR signalling, reducing inflammatory mediator release, and lowering gout attack frequency in patients with diabetes.

The clinical studies considered for evaluating the therapeutic effects of Metformin were as follows: six for RA, three studies for SLE, and one for SS and gout. In SLE, metformin supplementation showed promise in reducing glucocorticoid dosage, disease flares, and prednisone exposure while restoring the Th17/Treg cell balance. Similarly, in RA, Metformin exhibited diverse benefits, including decreased admission rates, improved treatment response rates, reduced disease activity, and enhanced quality of life for patients. The treatment was associated with a decreased risk for RA and showed a negative causal association with RA pathogenesis, suggesting its potential inhibitory role in disease development.

Since limited literature was available, preclinical and clinical findings were considered for evaluating the effects of SGLT2 inhibitors in rheumatic diseases. The review encompassed four articles on SLE, five on gout, and one on lupus nephritis. Zhao et al. demonstrated the renoprotective effect of empagliflozin in lupus mice, showcasing reductions in proteinuria and stable eGFR in lupus nephritis patients. A multi-centre study by Jorge et al. highlighted the cardiac and renal benefits of SGLT2 inhibitors in SLE and lupus nephritis patients, emphasising reduced risks of major cardiovascular events and renal progression. Lo et al. corroborated these findings in SLE patients with T2DM, reporting lower risks of acute kidney injury and heart failure with SGLT2 inhibitor usage. Petri et al. observed improved urine protein/creatinine ratio and stable eGFR in SLE patients taking SGLT2 inhibitors. Phase I/II trials by Wang et al. affirmed the safety profile of dapagliflozin in adult SLE patients and the associated favourable metabolic and renal outcomes. In gout patients with T2DM, Wei et al. found lower risks of recurrent gout flares and mortality with SGLT2 inhibitors compared to other medications. Fralick et al. and subsequent studies by Lund, Chung, and Zhou et al. further supported the association between SGLT2 inhibitor use and reduced gout incidence, suggesting their potential as therapeutic agents in managing rheumatic diseases.

The present narrative review holds significant relevance as no single study provides a comprehensive overview of the therapeutic effects of metformin or SGLT2 inhibitors in various rheumatologic diseases, integrating evidence from preclinical and clinical studies. The review’s strengths lie in its broad coverage of diseases and the integration of preclinical and clinical study findings, which bridge the gap between laboratory research and clinical practice. However, limitations include the inability to conduct a systematic review and meta-analysis due to the heterogeneity of available literature studies. Furthermore, the broad scope of the review limits the generalisation of the findings and extrapolating conclusions beyond the scope of individual studies.

## CONCLUSION

The review confirms the multifaceted potential of Metformin as an adjunctive therapy in autoimmune rheumatologic conditions, presenting opportunities for further exploration and clinical application to enhance patient outcomes. Limited literature exists regarding the clinical effects of SGLT2 inhibitors in rheumatic diseases. Moreover, the review emphasises the need for additional research, including head-to-head comparisons, to comprehend the precise mechanisms of action of metformin and SGLT2 inhibitors and optimise their application in personalised medicine for autoimmune conditions.

## Figures and Tables

**Supplementary Table 1.** Tally of articles retrieved from various databases following the search.

**Search terms**	**No of articles**
SGLT2 AND Immunomodulatory effect	454
Metformin AND Immunomodulatory effect	605
Metformin AND Rheumatic diseases	151
Metformin AND Autoimmune diseases	551
Metformin AND Connective tissue disorders	76
SGLT2inhibitors AND Rheumatic diseases	41
SGLT2inhibitors AND Autoimmune diseases	354
SGLT2 inhibitors AND Connective tissue disorders	27
SGLT2 inhibitors AND Rheumatoid Arthritis	34
SGLT2 inhibitors AND Lupus	48
SGLT2 inhibitors AND Psoriatic Arthritis	25
SGLT2 inhibitors AND Ankylosing Spondylitis	26
SGLT2 inhibitors AND Sjögren’s syndrome	24
SGLT2 AND Gout	52
SGLT2 AND Scleroderma	20
SGLT2 AND Infectious Arthritis	3
SGLT2 AND Juvenile Idiopathic Arthritis	2
SGLT2 AND Polymyalgia rheumatica	4
Metformin AND Rheumatoid Arthritis	71
Metformin AND Lupus	106
Metformin AND Psoriatic Arthritis	7
Metformin AND Ankylosing Spondylitis	9
Metformin AND Sjögren’s syndrome	17
Metformin AND Gout	42
Metformin AND Scleroderma	18
Metformin AND Infectious Arthritis	2
Metformin AND Juvenile Idiopathic Arthritis	3
Metformin AND Polymyalgia rheumatica	2
Canagliflozin AND Rheumatoid Arthritis	8
Canagliflozin AND Lupus	4
Canagliflozin AND Psoriatic Arthritis	1
Canagliflozin AND Ankylosing Spondylitis	3
Canagliflozin AND Sjögren’s syndrome	2
Canagliflozin AND Gout	25
Canagliflozin AND Scleroderma	6
Canagliflozin AND Infectious Arthritis	3
Canagliflozin AND Juvenile Idiopathic Arthritis	3
Canagliflozin AND Polymyalgia rheumatica	5
Dapagliflozin AND Rheumatoid Arthritis	4
Dapagliflozin AND Lupus	6
Dapagliflozin AND Psoriatic Arthritis	4
Dapagliflozin AND Ankylosing Spondylitis	3
Dapagliflozin AND Sjögren’s syndrome	2
Dapagliflozin AND Gout	9
Dapagliflozin AND Scleroderma	6
Dapagliflozin AND Infectious Arthritis	8
Dapagliflozin AND Juvenile Idiopathic Arthritis	1
Dapagliflozin AND Polymyalgia rheumatica	2
Empagliflozin AND Rheumatoid Arthritis	1
Empagliflozin AND Lupus	4
Empagliflozin AND Psoriatic Arthritis	2
Empagliflozin AND Ankylosing Spondylitis	1
Empagliflozin AND Sjögren’s syndrome	1
Empagliflozin AND Gout	7
Empagliflozin AND Scleroderma	2
Empagliflozin AND Infectious Arthritis	1
Empagliflozin AND Juvenile Idiopathic Arthritis	2
Empagliflozin AND Polymyalgia rheumatica	2

## References

[1] Elders MJ. The increasing impact of arthritis on public health. J Rheumatol Suppl 2000 Oct;60:6–8. 11032095

[2] Urwin M Symmons D Allison T Brammah T Busby H Roxby M Estimating the burden of musculoskeletal disorders in the community: the comparative prevalence of symptoms at different anatomical sites, and the relation to social deprivation. Ann Rheum Dis 1998 Nov;57(11):649–55. 9924205 10.1136/ard.57.11.649 PMC1752494

[3] Mehta P Gasparyan AY Zimba O Kitas GD Yessirkepov M. Interplay of diabetes mellitus and rheumatic diseases amidst the COVID-19 pandemic: influence on the risk of infection, outcomes, and immune responses. Clin Rheumatol 2022 Dec;41(12):3897–913. 36076125 10.1007/s10067-022-06365-y PMC9458477

[4] Tian Z Mclaughlin J Verma A Chinoy H Heald AH. The relationship between rheumatoid arthritis and diabetes mellitus: a systematic review and meta-analysis. Cardiovasc Endocrinol Metab 2021 Feb 19;10(2):125–31. 34124603 10.1097/XCE.0000000000000244 PMC8189616

[5] Alobaidi S Alotaibi M Al-Zahrani N Al-Dhaheri F. (2021). Renal System and Rheumatology. In: Almoallim H. Cheikh M. (Eds.), Skills in Rheumatology. Singapore: Springer. Available from: http://www.ncbi.nlm.nih.gov/books/NBK585750/ [Accessed April 17, 2024]. 36315807

[6] Hill AJ Thomson RJ Hunter JA Traynor JP. The prevalence of chronic kidney disease in rheumatology outpatients. Scott Med J 2009 May;54(2):9–12. 19530494 10.1258/rsmsmj.54.2.9

[7] Atzeni F Nucera V Gerratana E Fiorenza A Gianturco L Corda M Cardiovascular Consequences of Autoimmune Rheumatic Diseases. Curr Vasc Pharmacol 2020;18(6):566–79. 31985379 10.2174/1570161118666200127142936

[8] Hollan I Meroni PL Ahearn JM Cohen Tervaert JW Curran S Goodyear CS Cardiovascular disease in autoimmune rheumatic diseases. Autoimmun Rev 2013 Aug;12(10):1004–15. 23541482 10.1016/j.autrev.2013.03.013

[9] Nurmohamed MT. Cardiovascular risk in rheumatoid arthritis. Autoimmun Rev 2009 Jul;8(8):663–7. 19393192 10.1016/j.autrev.2009.02.015

[10] Myasoedova E Crowson CS Turesson C Gabriel SE Matteson EL. Incidence of extraarticular rheumatoid arthritis in Olmsted County, Minnesota, in 1995–2007 versus 1985–1994: a population-based study. J Rheumatol 2011 Jun;38(6):983–9. 21459933 10.3899/jrheum.101133 PMC3193155

[11] Fausto S Marco DC Marina C Sonia F Alessandro C Marwin G. The impact of different rheumatic diseases on health-related quality of life: a comparison with a selected sample of healthy individuals using SF-36 questionnaire, EQ-5D, and SF-6D utility values. Acta Biomed 2018;89(4):541–57. 10.23750/abm.v89i4.7298 PMC6502108 30657123

[12] Soriano ER. Current Status and Future Challenges in the Treatment of Rheumatic Diseases. FrontDrug Saf Regul 2022;2:881556.

[13] Qin C Diaz-Gallo LM Tang B Wang Y Nguyen TD Harder A Repurposing antidiabetic drugs for rheumatoid arthritis: results from a two-sample Mendelian randomization study. Eur J Epidemiol 2023;38(7):809–19. 37052755 10.1007/s10654-023-01000-9 PMC10276071

[14] Padda IS Mahtani AU Parmar M. Sodium-Glucose Transport Protein 2 (SGLT2) Inhibitors. In: StatPearls [Internet]. Treasure Island (FL): StatPearls Publishing; 2024 [cited 2024 Feb 29]. Available from: http://www.ncbi.nlm.nih.gov/books/NBK576405/35015430

[15] Oakes E Ellrodt J Choi M Guan H Costenbader K. Prescribing Patterns of SGLT2 Inhibitors for Patients with Autoimmune Rheumatic Disease [abstract]. Arthritis Rheumatol 2022;74 (suppl 9).

[16] Kim JW Choe JY Park SH. Metformin and its therapeutic applications in autoimmune inflammatory rheumatic disease. Korean J Intern Med 2022 Jan;37(1):13–26. 34879473 10.3904/kjim.2021.363 PMC8747910

[17] Kang KY Kim YK Yi H Kim J Jung HR Kim IJ Metformin down-regulates Th17 cell differentiation and attenuates murine autoimmune arthritis. Int Immunopharmacol 2013 May;16(1):85–92. 23557965 10.1016/j.intimp.2013.03.020

[18] Son HJ Lee J Lee SY Kim EK Park MJ Kim KW Metformin attenuates experimental autoimmune arthritis through reciprocal regulation of Th17/Treg balance and osteoclastogenesis. Mediators Inflamm 2014;2014:973986. 25214721 10.1155/2014/973986 PMC4158168

[19] Yan H Zhou HF Hu Y Pham CTN. Suppression of experimental arthritis through AMP-activated protein kinase activation and autophagy modulation. J Rheum Dis Treat 2015 Feb 28;1(1):5. 26120598 10.23937/2469-5726/1510005 PMC4479345

[20] Kim EK Lee SH Lee SY Kim JK Jhun JY Na HS Metformin ameliorates experimental-obesity-associated autoimmune arthritis by inducing FGF21 expression and brown adipocyte differentiation. Exp Mol Med 2018 Jan 26;50(1):e432. 29371695 10.1038/emm.2017.245 PMC5799802

[21] Chen K Lin ZW He SM Wang CQ Yang JC Lu Y Metformin inhibits the proliferation of rheumatoid arthritis fibroblast-like synoviocytes through IGF-IR/PI3K/AKT/mTOR pathway. Biomed Pharmacother 2019 Jul;115:108875. 31028998 10.1016/j.biopha.2019.108875

[22] Chen Y Qiu F Yu B Chen Y Zuo F Zhu X Metformin, an AMPK Activator, Inhibits the Activation of FLSs but Promotes HAPLN1 Secretion. Mol Ther Methods Clin Dev 2020 Jun 12;17:1202–14. 32518807 10.1016/j.omtm.2020.05.008 PMC7275116

[23] Fan KJ Wu J Wang QS Xu BX Zhao FT Wang TY. Metformin inhibits inflammation and bone destruction in collagen-induced arthritis in rats. Ann Transl Med 2020 Dec;8(23):1565. 33437764 10.21037/atm-20-3042 PMC7791269

[24] Matsuoka Y Morimoto S Fujishiro M Hayakawa K Kataoka Y Suzuki S Metformin repositioning in rheumatoid arthritis. Clin Exp Rheumatol 2021;39(4):763–8. 32828146

[25] Park YH Jang YJ Choi Y Lee K Kim HJ Cho O Combination of LMT-28 and Metformin Improves Beneficial Anti-Inflammatory Effect in Collagen-Induced Arthritis. Pharmacology 2021;106(1–2):53–9. 32674107 10.1159/000507451

[26] Kim EK Min HK Lee SY Kim DS Ryu JG Na HS Metformin rescues rapamycin-induced mitochondrial dysfunction and attenuates rheumatoid arthritis with metabolic syndrome. Arthritis Res Ther 2020 Apr 10;22(1):77. 32276645 10.1186/s13075-020-02174-3 PMC7149912

[27] El-Sayyad SM Ali MA Kandil LS Ragab GM Abdelhamid Ibrahim SS. Metformin and omega-3 fish oil elicit anti-inflammatory effects via modulation of some dysregulated micro RNA expression and signalling pathways in experimentally induced arthritis. Int Immunopharmacol 2021 Mar;92:107362. 33453674 10.1016/j.intimp.2020.107362

[28] Gallagher L Cregan S Biniecka M Cunningham C Veale DJ Kane DJ Insulin-Resistant Pathways Are Associated With Disease Activity in Rheumatoid Arthritis and Are Subject to Disease Modification Through Metabolic Reprogramming: A Potential Novel Therapeutic Approach. Arthritis Rheumatol 2020 Jun;72(6):896–902. 31840936 10.1002/art.41190

[29] Makar N Elhawary AH Emam H Abo Ria N Shaaban EES. Possible Beneficial Effect of Metformin Alone or in Combination with Methotrexate in Rheumatoid Arthritis Induced Rat Model. Benha Med J 2020 May 1;37(1):143–54.

[30] Jhun J Lee S Kim SY Na HS Kim EK Kim JK Combination therapy with metformin and coenzyme Q10 in murine experimental autoimmune arthritis. Immunopharmacol Immunotoxicol 2016;38(2):103–12. 26681425 10.3109/08923973.2015.1122619

[31] Yang M Ding Y Wang Y Gu J Zhang B Wang H. Metformin regulates of Th17/treg cell balance and reduces hyperplastic synovium via activating AMPK and inhibiting mTOR in a collagen-induced arthritis rat model. Int J Clin Exp Med 2017 Jan 1;10:11479–87.

[32] Liang J Cai Y Zhang J Jing Z Lv L Zhang G Metformin Treatment Reduces the Incidence of Rheumatoid Arthritis: A Two-Sample Mendelian Randomized Study. J Clin Med 2023 Mar 23;12(7):2461. 37048545 10.3390/jcm12072461 PMC10095374

[33] Abdallah MS Alarfaj SJ Saif DS El-Naggar ME Elsokary MA Elsawah HK The AMPK modulator metformin as adjunct to methotrexate in patients with rheumatoid arthritis: A proof-of-concept, randomized, double-blind, placebo-controlled trial. Int Immunopharmacol 2021 Jun;95:107575. 33773207 10.1016/j.intimp.2021.107575

[34] Gharib M Elbaz W Darweesh E Ali Sabri N Shawki M. Efficacy and Safety of Metformin Use in Rheumatoid Arthritis: A Randomized Controlled Study. Front Pharmacol 2021 Sep 22;12. 10.3389/fphar.2021.726490 PMC8493211 34630103

[35] Lu CH Chung CH Lee CH Su SC Liu JS Lin FH Combination of COX-2 inhibitor and Metformin attenuates rate of admission in patients with rheumatoid arthritis and diabetes in Taiwan. Medicine (Baltimore) 2019 Oct 11;98(41):e17371. 31593087 10.1097/MD.0000000000017371 PMC6799465

[36] Naffaa ME Rosenberg V Watad A Tiosano S Yavne Y Chodick G Adherence to Metformin and the onset of rheumatoid arthritis: a population-based cohort study. Scand J Rheumatol 2020 May;49(3):173–80. 32208872 10.1080/03009742.2019.1695928

[37] Zhang L Zhou Y Jiang S Fan Y Huang J Xiao B Effects of metformin therapy on HMGB1 levels in rheumatoid arthritis patients. Eur J Med Res 2023 Nov 15;28(1):512. 37964313 10.1186/s40001-023-01476-x PMC10648365

[38] Yin Y Choi SC Xu Z Perry DJ Seay H Croker BP Normalization of CD4+ T Cell Metabolism Reverses Lupus. Sci Transl Med 2015 Feb 11;7(274):274ra18. 10.1126/scitranslmed.aaa0835 PMC5292723 25673763

[39] Kansurkar S Rai MK Misra DP Agarwal V. AB0167 Metformin has anti-inflammatory potential by reducing p-gp expression on pbmcs of patients with lupus. Ann Rheum Dis 2018 Jun 1;77(Suppl 2):1271–2.

[40] Sumantri S Hatta M Natzir R Rasyid H Rengganis I Massi MN Metformin improves FOXP3 mRNA expression through suppression of interferon-gamma levels in pristane-induced murine models of lupus. F1000Res 2021 Jun 11;9:342. 10.12688/f1000research.23471.1 PMC8327221 34386197

[41] Jang SG Lee J Hong SM Kwok SK Cho ML Park SH. Metformin enhances the immunomodulatory potential of adipose-derived mesenchymal stem cells through STAT1 in an animal model of lupus. Rheumatology 2020 Jun 1;59(6):1426–38. 31904843 10.1093/rheumatology/kez631

[42] Chen XC Wu D Wu HL Li HY Yang C Su HY Metformin improves renal injury of MRL/lpr lupus-prone mice via the AMPK/STAT3 pathway. Lupus Sci Med 2022 Apr;9(1):e000611. 35414608 10.1136/lupus-2021-000611 PMC9006817

[43] Sun F Geng S Wang H Wang H Liu Z Wang X Effects of Metformin on disease flares in patients with systemic lupus erythematosus: post hoc analyses from two randomized trials. Lupus Sci Med 2020 Oct;7(1):e000429. 33093216 10.1136/lupus-2020-000429 PMC7583791

[44] Wang H Li T Chen S Gu Y Ye S. Neutrophil Extracellular Trap Mitochondrial DNA and Its Autoantibody in Systemic Lupus Erythematosus and a Proof-of-Concept Trial of Metformin. Arthritis Rheumatol 2015 Dec;67(12):3190–200. 26245802 10.1002/art.39296

[45] Hao M Liang Z Jing X Liu XQ Gao C Li X AB0523 Metformin combined with conventional therapy increases absolute number of regulatory t cells in patients with systemic lupus erythematosus [abstract]. Ann Rheum Dis 2018 Jun 1;77(Suppl 2):1419–1419.

[46] Qin X Jiang T Liu S Tan J Wu H Zheng L Effect of Metformin on ossification and inflammation of fibroblasts in ankylosing spondylitis: An in vitro study. J Cell Biochem 2018 Jan;119(1):1074–82. 28696014 10.1002/jcb.26275

[47] Nath N Khan M Paintlia MK Hoda MN Giri S. Metformin Attenuated the Autoimmune Disease of the Central Nervous System in Animal Models of Multiple Sclerosis. J Immunol 2009 Jun 15;182(12):8005–14. 19494326 10.4049/jimmunol.0803563 PMC2965405

[48] Nascimento Da Conceicao V Sun Y Chai X Ambrus JL Mishra BB Singh BB. Metformin-induced activation of Ca2+ signaling prevents immune infiltration/pathology in Sjogren’s syndrome-prone mouse models. J Transl Autoimmun 2023 Sep 9;7:100210. 37711153 10.1016/j.jtauto.2023.100210 PMC10497794

[49] Wang CY Lai JN Liu CH Hu KC Sheu KL Wei JCC. Metformin Use Was Associated With Reduced Risk of Incidental Sjögren’s Syndrome in Patients With Type 2 Diabetes: A Population-Based Cohort Study. Front Med (Lausanne) 2021;8:796615. 35096887 10.3389/fmed.2021.796615 PMC8790021

[50] Tsuji G Hashimoto-Hachiya A Yen VH Takemura M Yumine A Furue K Metformin inhibits IL-1β secretion via impairment of NLRP3 inflammasome in keratinocytes: implications for preventing the development of psoriasis. Cell Death Discov 2020;6:11. 32194991 10.1038/s41420-020-0245-8 PMC7055596

[51] Wang Y Zhang S Liang Z Feng M Zhao X Qin K Metformin attenuates bleomycin-induced scleroderma by regulating the balance of Treg/Teff cells and reducing spleen germinal center formation. Mol Immunol 2019 Oct;114:72–80. 31344551 10.1016/j.molimm.2019.07.002

[52] Vazirpanah N Ottria A Linden M van der, Wichers CGK Schuiveling M Lochem E van mTOR inhibition by metformin impacts monosodium urate crystal–induced inflammation and cell death in gout: a prelude to a new add-on therapy? Ann Rheum Dis 2019 May 1;78(5):663–71. 30814053 10.1136/annrheumdis-2018-214656

[53] Veenstra F Verhoef LM Opdam M den Broeder AA Kwok WY Meek IL Effect of metformin use on clinical outcomes and serum urate in gout patients with diabetes mellitus: a retrospective cohort study. BMC Rheumatol 2022 May 31;6(1):27. 35637534 10.1186/s41927-022-00261-3 PMC9153141

[54] Zhao XY Li SS He YX Yan LJ Lv F Liang QM SGLT2 inhibitors alleviated podocyte damage in lupus nephritis by decreasing inflammation and enhancing autophagy. Ann Rheum Dis 2023 Oct 1;82(10):1328–40. 37487609 10.1136/ard-2023-224242

[55] Jorge A Zhou B McCormick N Yokose C Zhang Y Choi H. Sodium-Glucose Co-transporter-2 Inhibitors and the Risk of Cardiac and Renal Outcomes in Systemic Lupus Erythematosus [abstract]. Arthritis Rheumatol 2023;75(suppl 9).

[56] Wang H Li T Sun F Liu Z Zhang D Teng X Safety and efficacy of the SGLT2 inhibitor dapagliflozin in patients with systemic lupus erythematosus: a phase I/II trial. RMD Open 2022 Oct;8(2):e002686. 36288823 10.1136/rmdopen-2022-002686 PMC9615980

[57] Lo J Huang P Tsokos G Kyttaris V Costenbader K Ma K. Comparing Safety and Efficacy of Sodium-Glucose Cotransporter 2 Inhibitors versus Dipeptidyl Peptidase 4 Inhibitors in Patients with Systemic Lupus Erythematosus and Comorbid Type 2 Diabetes Mellitus [abstract]. Arthritis Rheumatol 2023;75(suppl 9).

[58] Michelle Petri Daniel Goldman Andrea Fava Larry Magder. 1490: Early Experience with SGLT2i in Systemic Lupus Erythematosus [abstract]. ACR 2023.

[59] Wei J Choi HK Dalbeth N Li X Li C Zeng C Gout Flares and Mortality After Sodium-Glucose Cotransporter-2 Inhibitor Treatment for Gout and Type 2 Diabetes. JAMA Netw Open 2023 Aug 25;6(8):e2330885. 37624597 10.1001/jamanetworkopen.2023.30885 PMC10457713

[60] Fralick M Chen SK Patorno E Kim SC. Assessing the Risk for Gout With Sodium-Glucose Cotransporter-2 Inhibitors in Patients With Type 2 Diabetes: A Population-Based Cohort Study. Ann Intern Med 2020 Feb 4;172(3):186–94. 31931526 10.7326/M19-2610 PMC7217750

[61] Lund LC Højlund M Henriksen DP Hallas J Kristensen KB. Sodium-glucose cotransporter-2 inhibitors and the risk of gout: A Danish population based cohort study and symmetry analysis. Pharmacoepidemiol Drug Saf 2021 Oct;30(10):1391–5. 33881179 10.1002/pds.5252

[62] Chung MC Hung PH Hsiao PJ Wu LY Chang CH Wu MJ Association of Sodium-Glucose Transport Protein 2 Inhibitor Use for Type 2 Diabetes and Incidence of Gout in Taiwan. JAMA Netw Open 2021 Nov 1;4(11):e2135353. 34797368 10.1001/jamanetworkopen.2021.35353 PMC8605485

[63] Zhou J Liu X Chou OHI Li L Lee S Wong WT Lower risk of gout in sodium glucose cotransporter 2 (SGLT2) inhibitors versus dipeptidyl peptidase-4 (DPP4) inhibitors in type-2 diabetes. Rheumatology (Oxford) 2023 Apr 3;62(4):1501–10. 36066415 10.1093/rheumatology/keac509

